# Virchow node of an unusual aetiology – the experience of a clinical case

**DOI:** 10.1186/s13044-022-00128-2

**Published:** 2022-05-16

**Authors:** Marta Borges-Canha, João Sérgio-Neves, Inês Albuquerque, João Pacheco, Maria Manuel Silva, Ana Isabel Oliveira, Davide Carvalho

**Affiliations:** 1 Serviço de Endocrinologia, Diabetes e Metabolismo, Centro Hospitalar e Universitário de S. João, Porto, Portugal; 2 Faculdade de Medicina e Instituto de Investigação e Inovação em Saúde da Universidade do Porto, Porto, Portugal; 3 Serviço de Medicina Interna, Centro Hospitalar e Universitário de S. João, Porto, Portugal; 4 Serviço de Anatomia Patológica do Centro Hospitalar Universitário de S. João, Porto, Portugal

**Keywords:** Virchow node, Supraclavicular node, Ectopic thyroid

## Abstract

**Background:**

The cause of adult adenomegalies may be defiant. On the other hand, ectopic thyroid is a rare condition that happens in every 1:100000 to 300,000 of healthy individuals. Here, we present a case report that joins these two clinical rare and defiant challenges.

**Clinical case:**

Forty-seven-year-old woman, with known thyroid nodules for several years. She had no other relevant personal or familiar history. At our appointment she had no complaints. At the physical examination she had a palpable right thyroid nodule (previously known). The routine blood analysis showed normal thyroid function. The routine cervical ultrasonography showed no dimensional progression of the known thyroid nodules and identified a 31x18mm nodule at the left supraclavicular fossa. The patient underwent a cervical, thoracic, and abdominal computed tomography that exhibited no relevant findings, such as abdominal malignancies. The cytology of the nodule showed characteristics that were “compatible with a benign follicular nodule in ectopic thyroid tissue”.

**Conclusion:**

This is a rare case in which we incidentally found a follicular nodule in ectopic thyroid tissue in the left supraclavicular fossa. Given the rarity of the situation, clinical sense is the mainstay of treatment and follow-up.

## Background

The clinical evaluation of masses in the adult is frequently challenging. The detection of supraclavicular nodules is particularly worrying concerning that around 50% of the cases associate to malignancy. Right supraclavicular nodules are generally due to mediastinal, lung or oesophagus malignancies. Left supraclavicular nodules (when solitary is called Virchow node) are suspicious of abdominal malignancies [1].

On the other hand, ectopic thyroid gland is found in one in every 100,000 to 300,000 of healthy individuals, mainly females, being the most common location the base of the tongue. It has also been described in many other sites, such as sublingual, submandibular region, oesophagus, diaphragm, duodenum, and vagina [2–5]. It is usually asymptomatic and frequently is not diagnosed and the true incidence is not known; symptoms as obstruction and bleeding may occur according to the location [6, 7]. The cause of ectopic thyroid is not completely understood [6]. Thyroid is the first endocrine gland appearing during the embryonal life. This gland arises at the 3rd week of gestation and descends from caudal to the tongue bud, to the neck and finally to its pretracheal location [7, 8]. It is believed that usually the ectopic thyroid tissue arises from embryologic development, differentiation, and migration anomalies [9].

Here, we present a case report that joins these two clinical rare and defiant challenges.

## Case report/case presentation

This is the case of a forty-seven-year-old woman. She was sent to the Endocrinology appointment to follow-up her known thyroid nodules. The patient reported that she had multiple thyroid nodules that were known for many years and that one of the nodules had already been submitted to fine-needle aspiration (follicular adenoma). She did not refer any other relevant personal or familiar history. She had no complaints or recent changes in her health status (namely, she denied weight loss, nausea, loss of appetite or feeling unwell). At the physical examination she had a palpable right thyroid nodule (previously known and described); she had normal blood pressure (127/80 mmHg) and heart rate (77 bpm) and had a normal body mass index.

**Fig. 1:**
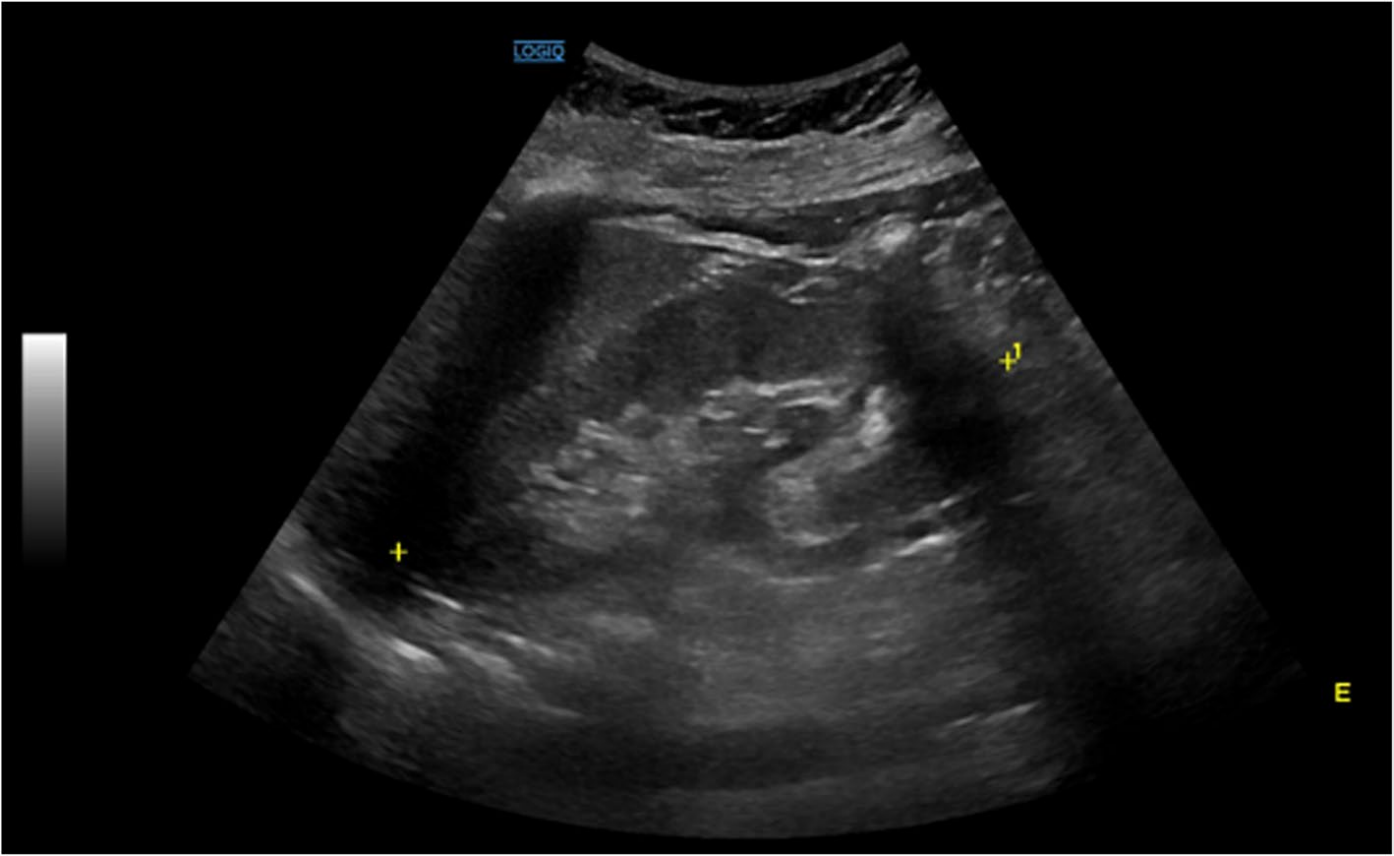
Ultrasonography showing an unspecific 31x18mm solid nodule at the left supraclavicular fossae

**Fig. 2:**
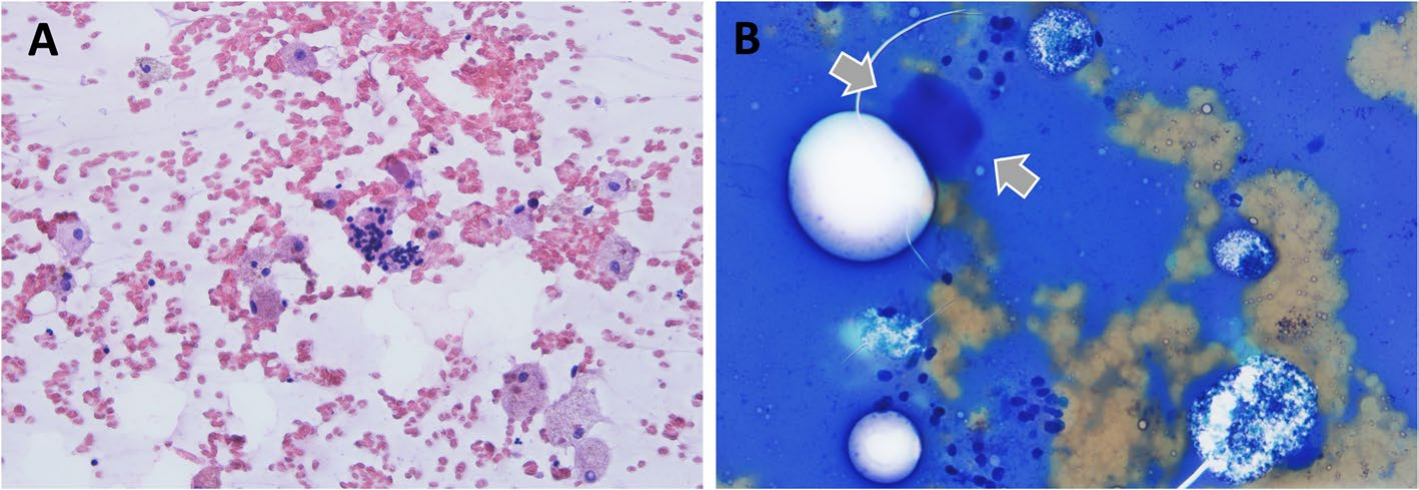
Cytologic evaluation (fine-needle aspiration of the supraclavicular mass). Here we can see numerous macrophages and epithelial cells arranged in sheets or as isolated cells. The nuclei of the epithelial cells are round and hyperchromatic. **A-** hematoxylin-eosin stain, 200x. **B-** giemsa stain, 400x. Arrows are showing thick colloid, which was seen focally

Table 1 displays some laboratory workup that shows thyroid function within the reference range. The routine cervical ultrasonography showed no dimensional progression of the known thyroid nodules; however, this exam identified an unspecific 31x18mm solid nodule at the left supraclavicular fossae (Fig. 1). Due to the concern raised with this mass, we decided to perform an ultrasound guided needle biopsy. The cytology (Fig. 2) showed characteristics that were “compatible with a benign follicular nodule in ectopic thyroid tissue”. Additionally, the patient underwent a cervical, thoracic, and abdominal computed tomography that excluded other possible causes to the incidental finding, such as abdominal malignancies. According to patients’ preference and multidisciplinary team, we agreed on active surveillance.

**Table 1:** Follow-up blood analysis

Parameter	Result	Normal value
**Haemoglobin**	13.0	12.0 to 16.0 g/dL
**Free T4**	0.90	0.70 to 1.48 ng/dL
**TSH**	0.44	0.35 to 4.94 UI/mL
**Creatinine**	0.64	0.51 to 0.95 mg/dL
**C-reactive protein**	1.4	< 3.0 mg/L

## Discussion and conclusion

Here we present a case of ectopic thyroid tissue with a follicular nodule, presenting as an incidentally found left supraclavicular mass in a woman with thyroid nodules and no other relevant past or familiar history.

The cause of adult adenomegalies is sometimes straightforward, considering anamnesis and physical examination. However, it may be defiant and is, sometimes, worrisome. When found, the Virchow node must be studied given its strong association with malignancies; for this reason, it is sometimes called sentinel node. It is most frequently a metastasis of an abdominal or thoracic carcinoma, mainly from a gastric carcinoma [1, 4].

On the other hand, ectopic thyroid is a rare condition for which molecular mechanisms are largely unknown; however, there have been studies showing mutations in genes expressed in the developing thyroid [6, 10]. As far as we are concerned, there is only one case reporting ectopic thyroid tissue in the same location; nevertheless, it was associated with seeding from a previous surgical procedure [9].

Given the rarity of the situation, clinical sense is the mainstay of treatment and follow-up. Generally, authors agree that the age and status of the patient, location of the finding, local symptoms and cytology should be considered in the decision [6]. The options can be active surveillance, surgical resection, or suppressive therapy (namely in poor surgical candidates in whom the size is a problem) [6]. In this case, according to patients’ preference, we agreed on active surveillance.

## Data Availability

Data sharing is not applicable to this article as no datasets were generated or analysed during the current study.
